# Supplementary material for: Dynamic cortical representations of perceptual filling-in for missing acoustic rhythm
Source: Sci Rep. 2017 Dec 13;7:17536. doi: 10.1038/s41598-017-17063-0 (PMC5727537; doi:10.1038/s41598-017-17063-0)
Supplement: Supplementary file 1 — Supplementary Information [file 41598_2017_17063_MOESM1_ESM.pdf]

Supplementary Information for

Dynamic cortical representations of perceptual filling-in for missing acoustic rhythm

Francisco Cervantes Constantino[1*], Jonathan Z. Simon[1,2,3,4†]

[1]Program in Neuroscience and Cognitive Science, [2]Department of Electrical and Computer Engineering, [3]Department of Biology, [4]Institute for Systems Research; University of Maryland, College Park, MD 20742, USA. *Present address: Centro de Investigación Básica en Psicología, Universidad de la República, Montevideo 11200, Uruguay. †Corresponding author jzsimon@umd.edu

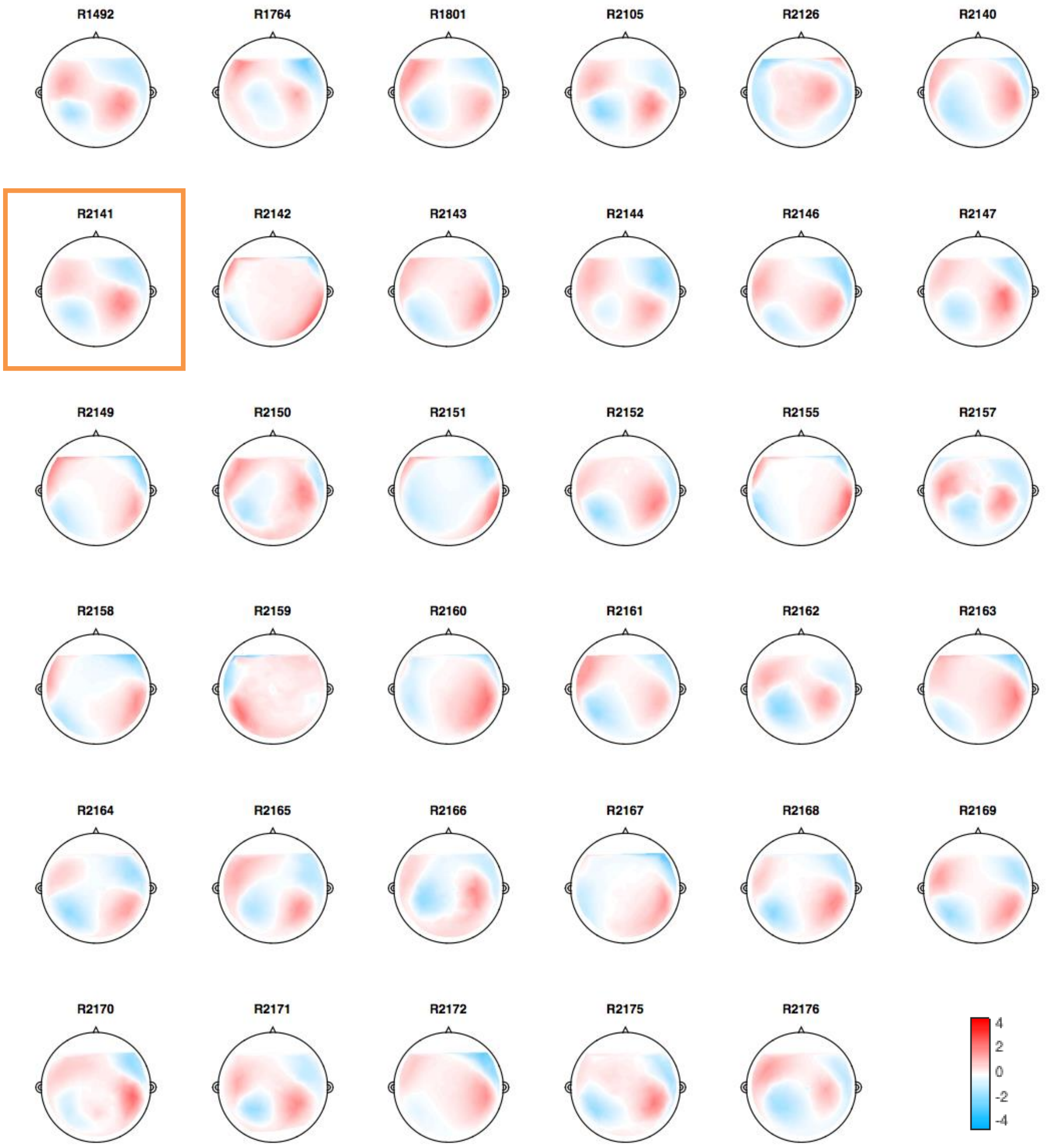

Supplementary Figure S1. Spatial filters associated with auditory steady-state responses. Spatial filters were obtained from participant datasets using responses from the unmasked acoustic pulse train only; the magnetic field distribution corresponding to that filter is displayed for each subject (N=35). The procedure constructs a fixed virtual MEG sensor on the basis of the most reproducible component of each participant's aSSR; neural dynamics during noise probes are investigated using this virtual sensor. The large majority of the field distributions are consistent with MEG evoked potentials originating from bilateral auditory cortex. The distribution from the representative subject in Fig. 1 is highlighted. Units are in z-scores.

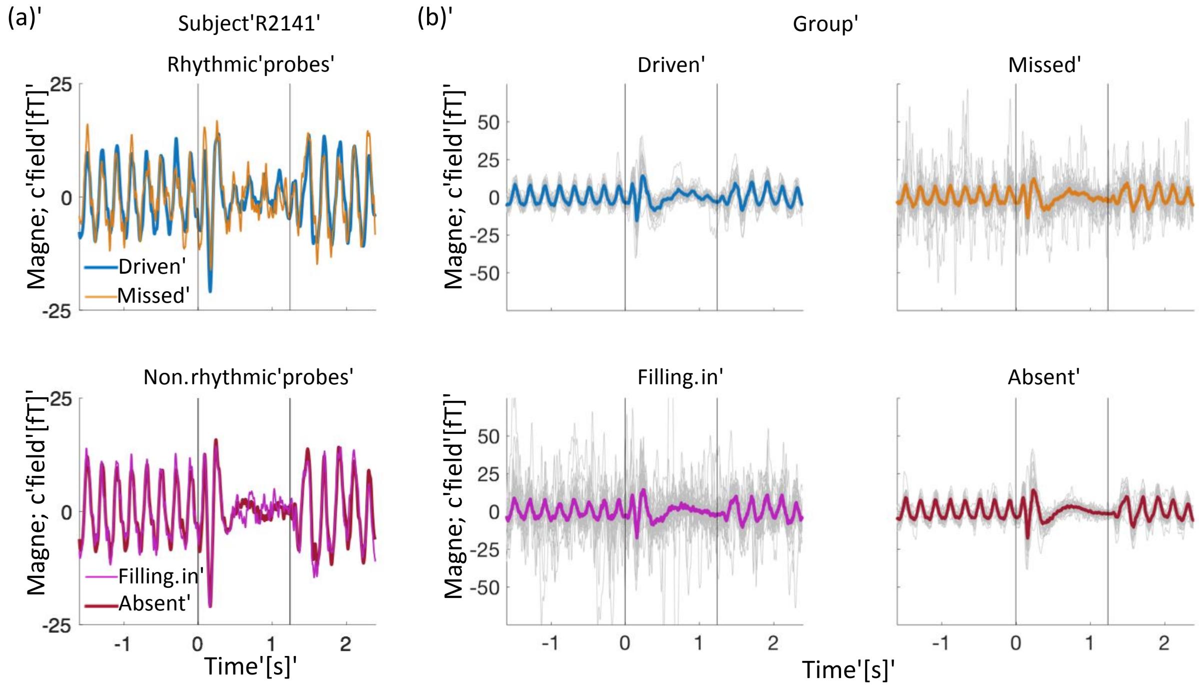

Supplementary Figure S2. Neural representations of a rhythmic pattern embedded in noise. (**a**) Representative stimulus-locked neural activity as measured by virtual MEG sensor. After a transient noise-onset response, the acoustic presence of a rhythmic pattern (top) may elicit an auditory steady-state response (aSSR) weaker in magnitude relative to baseline levels; (bottom) the acoustic absence of the target rhythmic pattern entails a similar noise-specific onset response, but an apparent lack of the aSSR depending on perception. (**b**) Median data across subjects with color convention as in as in (A); grey indicates individual subjects.

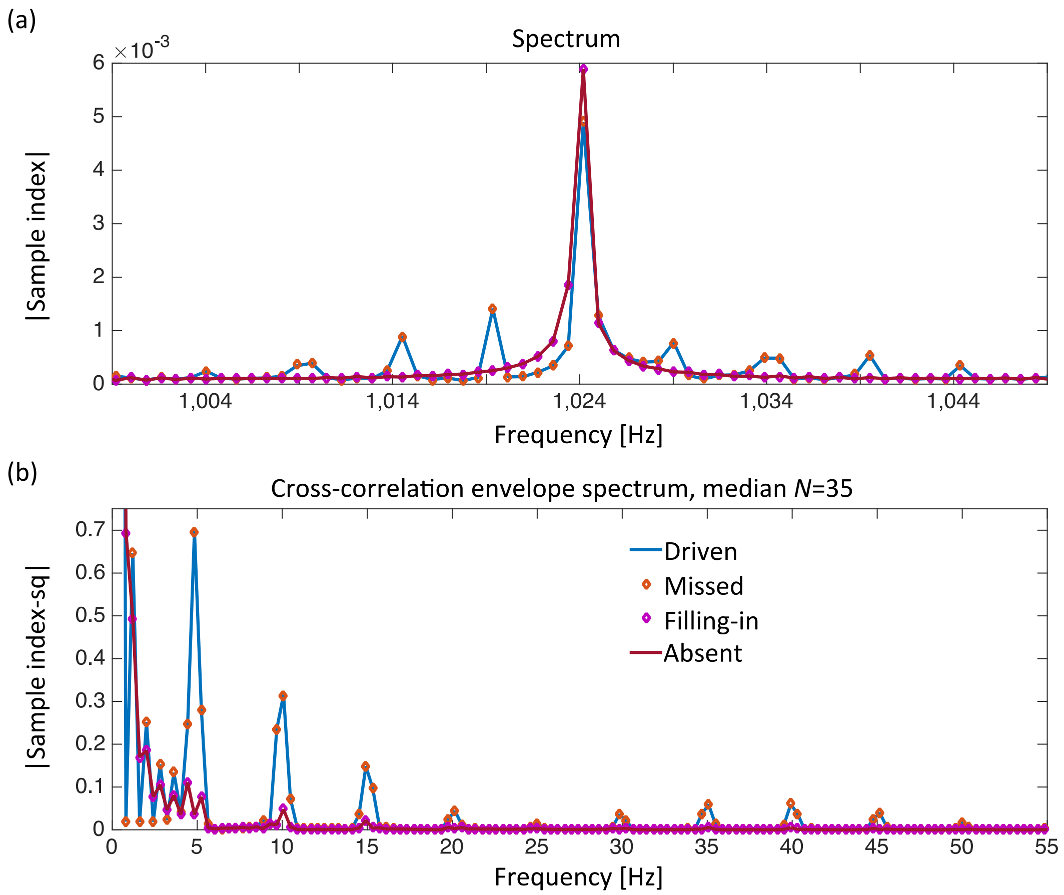

Supplementary Figure S3. No systematic acoustic influence of ambiguous perception of stimuli. (**a**) Spectral analysis of all rhythmic [respectively, non-rhythmic] noise probes in the experiment, shows that as per stimuli design, spectral content in probes appears virtually identical regardless of a listener's posterior report on their rhythmic [non-rhythmic] content. Spectra predominantly feature the 1024 Hz tone carrier, and FM interactions where expected (color code in (B)). (**b**) To assess for unforeseen random temporal modulations appearing systematically in the probe distributions, each probe trial was cross-correlated with a stimulus segment consisting of the basic pulse train without noise. 5 Hz modulations in the cross-correlation envelope are observed only when the pulse train was present were expected (i.e. rhythmic probes), because signal similarity peaks at periodic lags. Between identical-acoustics probe partitions, tests for pairwise differences with mean different than zero were rejected (paired-sample *t*-tests; rhythmic versus missed, p=0.85; filling-in versus absent, p=0.84).
